# A brain‐derived tau oligomer polymorph is associated with cognitive resilience to Alzheimer's disease

**DOI:** 10.1002/alz.70550

**Published:** 2025-08-18

**Authors:** Michela Marcatti, Batbayar Tumurbaatar, Wen Ru Zhang, Pietro Scaduto, Jutatip Guptarak, Shrinath Kadamangudi, Regan J. Schuetze, Anna Fracassi, Rakez Kayed, Giulio Taglialatela

**Affiliations:** ^1^ Mitchell Center for Neurodegenerative Disease, Department of Neurology The University of Texas Medical Branch at Galveston Galveston Texas USA; ^2^ Moody Brain Health Institute The University of Texas Medical Branch Galveston Texas USA

**Keywords:** Alzheimer's disease, brain‐derived tau oligomers, cognitive resilience, non‐demented with Alzheimer's neuropathology, tau polymorphs

## Abstract

**INTRODUCTION:**

Misfolded tau can assemble into oligomers that adopt distinct conformations, referred to as polymorphs, each with unique biochemical and pathological properties. These tau polymorphs are thought to influence disease progression in Alzheimer's disease (AD) and related disorders. Interestingly, some individuals with AD pathology remain cognitively intact (non‐demented with Alzheimer's neuropathology [NDAN]), suggesting potential differences in tau polymorph profiles.

**METHODS:**

Brain‐derived tau oligomers (BDTOs) were isolated from *post mortem* hippocampi of AD and NDAN individuals. Their biophysical, biochemical, and functional properties were assessed via protease digestion, immunocharacterization, atomic force microscopy, tau seeding in biosensor cells, hippocampal slice electrophysiology, and SH‐SY5Y toxicity assays.

**RESULTS:**

NDAN‐BDTOs exhibited protease resistance, different conformational profiles, formed larger aggregates, preserved synaptic function, and reduced neuronal toxicity compared to AD‐BDTOs.

**DISCUSSION:**

The data suggests that a structurally stable yet less toxic tau polymorph in NDAN may underlie cognitive resilience, supporting the therapeutic relevance of targeting specific tau polymorphs.

**Highlights:**

- Non‐demented with Alzheimer's neuropathology (NDAN) brain‐derived tau oligomers (BDTOs) exhibit distinct, more stable structural polymorphs compared to those from Alzheimer's disease (AD).
- NDAN‐BDTOs are larger, more protease resistant, and less toxic in cell and hippocampal slice models.
- Both NDAN‐ and AD‐BDTOs seed tau aggregation, but NDAN‐BDTOs promote formation of less toxic assemblies.
- Structural stability of NDAN‐BDTOs may contribute to cognitive resilience despite AD pathology.
- Mimicking non‐toxic tau polymorphs could represent a novel therapeutic strategy for AD.

## 1 BACKGROUND

Alzheimer's disease (AD) is a progressive neurodegenerative disorder, clinically characterized by cognitive decline and memory dysfunction.^1^ In AD, tau and amyloid beta (Aβ) misfold and aggregate into small, soluble oligomers, which accumulate and eventually form plaques and tangles—the histopathological hallmarks of the disease.^2^ Robust evidence suggests that Aβ and tau oligomers (AβO and TauO) are the most toxic species driving AD pathology.^3^, ^4^, ^5^, ^6^ In addition to abnormal protein aggregates, AD patients exhibit synaptic loss, mitochondrial dysfunction, neuroinflammation, and disrupted calcium homeostasis.^7^ The mechanisms underlying these changes and their relevance to cognitive decline are areas of ongoing research. Intriguingly, some individuals maintain cognitive integrity despite exhibiting tau tangles and amyloid plaques comparable to symptomatic AD patients. These individuals, classified as A+T+N− based on amyloid (A), tau (T), and neurodegeneration (N) biomarkers, will be referred to as “non‐demented with AD neuropathology” (NDAN) in this study.^8^ We previously reported that NDAN subjects exhibit a distinct synaptic proteomic profile and mitochondrial RNA (miRNA) regulation.^9^ Additionally, NDAN individuals showed increased neural stem cell numbers in the hippocampus,^10^ preserved antioxidant defenses,^11^ and autophagy responses.^8^ Moreover, we demonstrated that synapses from NDAN individuals are resistant to the toxic binding of AβO and TauO^12^, ^13^ and exhibit remodeling processes associated with the preservation of synaptic integrity and cognitive functions despite the presence of AD pathology.^14^

While neurofibrillary tangles have long been considered a hallmark of AD, recent evidence highlights soluble TauO as the key drivers of pathology due to their higher toxicity and ability to seed further tau misfolding.^15^, ^16^, ^17^, ^18^, ^19^, ^20^, ^21^ Notably, TauO exhibit structural heterogeneity, forming distinct polymorphs, some of which behave as strains, defined by their unique biochemical, biophysical, and pathological properties.^22^, ^23^ These tau polymorphs are hypothesized to underlie the pathological and clinical heterogeneity observed across tauopathies, including AD, progressive supranuclear palsy (PSP), and dementia with Lewy bodies (DLB).^24^, ^25^, ^26^ Structural differences in tau polymorphs may influence their stability, aggregation and seeding propensity, and neurotoxic potential, ultimately shaping disease progression and phenotype.

Building on these previous findings, we isolated brain‐derived tau oligomers (BDTOs) from *post mortem* brains of age‐matched AD patients and NDAN individuals to evaluate whether differences in the structural and functional properties of TauO polymorphs contribute to the cognitive resilience observed in NDAN, despite the presence of neurofibrillary tangles and Aβ plaques typically associated with clinically manifest AD. Consequently, through a series of studies, including immunoprecipitation, atomic force microscopy, proteinase digestion, electrophysiology, and cytotoxicity assays, we revealed distinct structural and functional differences between the BDTOs from AD and NDAN individuals. Notably, the BDTOs associated with NDAN exhibited reduced cytotoxicity and synaptotoxicity, key features of cognitive resilience.

## 2 METHODS

### 2.1 Human subjects and brain tissues

*Post mortem* brain tissues were obtained from the Oregon Brain Bank at Oregon Health and Science University (OHSU), in Portland, Oregon (USA). Male and female donor subjects were enrolled and clinically evaluated in studies at the National Institutes of Health–sponsored Layton Aging and Alzheimer's Disease Center (ADC) at OHSU, in accordance with protocols that were approved by the OHSU Institutional Review Board (IRB). Informed consent was obtained from all participants prior to their enrolment in the studies at the ADC. Subjects were participants in brain aging studies at the ADC and received annual neurological and neuropsychological evaluations, with a Clinical Dementia Rating (CDR) assigned by an experienced clinician. Hippocampal frozen tissues were dissected to include CA1, 2, 3, 4, subiculum, and all the dentate gyrus. The stratum radiatum and overlying external plexiform layer are included as these areas were difficult to dissect out. A neuropathological assessment was performed at autopsy, and in compliance with IRB‐approved protocols. A neuropathologist scored autopsy brain tissue for Aβ plaques and neurofibrillary tangles, according to standardized Consortium to Establish a Registry for Alzheimer's Disease criteria and Braak staging.^27^ Participants were classified as AD when possessing a National Institute for Neurological and Communicative Disorders and Stroke—Alzheimer's Disease and Related Disorder Association diagnostic criteria for clinical AD (CDR), including a Mini‐Mental State Examination^28^ (MMSE) score < 10. NDAN cases displayed little to no cognitive impairment (MMSE ≥ 27), though autopsy revealed presence of amyloid plaques and neurofibrillary tangles comparable to fully symptomatic AD (Table 1). Donor subject samples were de‐identified by ADC prior to being provided to the University of Texas Medical Branch (UTMB), so that no approval was required from the UTMB IRB under CFR §46.101(a).

**RESEARCH IN CONTEXT**

1. **Systematic review**: We reviewed literature using traditional sources, including PubMed, to identify studies on tau oligomer polymorphs, their role in Alzheimer's disease (AD), and the phenomenon of cognitive resilience in NDAN (non‐demented with Alzheimer's neuropathology) individuals. While numerous reports characterize tau oligomer toxicity in AD, few directly compare tau species from AD and NDAN brains to assess polymorph‐specific features.
2. **Interpretation**: Our data suggest that tau oligomers from NDAN brains differ structurally and functionally from those in AD, reflecting a stable but less pathogenic polymorph. These findings support the idea that polymorph variation may influence clinical outcomes despite similar pathological loads.
3. **Future directions**: Further research should investigate how tau polymorph identity relates to clinical outcomes and whether specific polymorph signatures can be detected in accessible biofluids. Determining the mechanisms that give rise to less toxic strains may uncover new therapeutic avenues to prevent or slow cognitive decline in AD.

**TABLE 1:** List of individuals used in this study.

Case	Diagnosis	Age	Sex	PMI, h	Braak
2312	AD	87	F	2.5	6
2316	AD	83	M	13	5
2374	AD	91	M	24	6
2359	AD	95	M	14	6
2491	NDAN	82	M	17	4
2556	NDAN	93	M	12	4
2980	NDAN	98	F	4.75	4
3178	NDAN	93	M	10	3

Abbreviations: AD, Alzheimer's disease; h, hours; NDAN, non‐demented with Alzheimer's neuropathology; PMI, *post mortem* interval.

### 2.2 Brain‐derived tau oligomers immunoprecipitation

To isolate BDTOs from AD and NDAN subjects, phosphate‐buffered saline (PBS) soluble human brain homogenate was prepared in accordance with protocols previously described.^16^ Briefly, 250 mg of hippocampal tissue from each of four subjects (AD cases: #2312, 2316, 2374, 2539; NDAN cases: #2491, 2556, 2980, 3178) were pooled to obtain a total of 1 g, and subsequently homogenized in 1X PBS supplemented with protease and phosphatase inhibitor cocktails (Roche, cat#:11836153001 and cat#:4906837001, respectively) at 1:5 (w/v) ratio and incubated for 20 minutes at 4°C. The samples were centrifuged at 10,000 × g for 10 minutes at 4°C, and supernatants were collected and aliquoted. The aliquots not used for immunoprecipitation were snap‐frozen and stored at −80°C until needed. BDTOs were immunoprecipitated (IP) with a tau‐oligo specific T18 antibody (Lo Cascio, Kayed R, 2020) using Pierce Co‐Immunoprecipitation Kit (Thermo Scientific, cat#26149). Briefly, 100 µL of AminoLink plus resin was coupled with 75 µg of affinity‐purified T18 antibody, incubated with 250 µL of PBS‐soluble fraction at 4°C for overnight. Sample‐antibody complex was washed three times with 200 µL of IP Lysis/Wash buffer, then eluted using 0.1 M glycine, pH 2.8. The pH of each eluted fraction was neutralized with 1 M Tris, pH 8.0. Total protein concentration was determined using Gene5 Microspot (BioTek). To validate the success of the IP samples were loaded on a 4% to 20% Criterion TGX Precast Gel (BioRad) followed by 45 minutes transfer to Amersham Protran nitrocellulose transfer membranes (GE Healthcare Life Sciences) at 85 V at 4°C. The membrane was blocked with 2% albumin (BSA) solution for 1 hour at room temperature (RT) and probed with the recombinant monoclonal anti‐human Tau antibody ([EPR2396[2]; 1:1000; Abcam, cat#: ab109392) overnight (ON) at 4°C. After incubation, the membrane was washed three times with 1 × tris‐buffered saline (TBS)/0.05%Tween solution (10 minutes each) and incubated 1 hour at RT with an anti‐rabbit immunoglobulin G (IgG), horseradish peroxidase (HRP)‐linked secondary antibody (1:5000; Cell Signaling cat#: 7074, RRID:AB_2099233). ECL Western Blotting Detection Reagents (Cytiva, cat#: RPN2209) was used for signal detection.

### 2.3 Immuno‐characterization of BDTOs from AD and NDAN subjects

To assess the immunoreactivity profiles of BDTOs from AD and NDAN individuals, eluates from IP_E1_ and IP_E2_ fractions were loaded onto 4% to 20% Criterion TGX Precast Gels (Bio‐Rad) and transferred for 45 minutes at 85 V at 4°C to Amersham Protran nitrocellulose membranes (GE Healthcare). Membranes were blocked for 1 hour at RT in 2% BSA in 1× TBS and ON at 4°C with one of the following primary antibodies at 1:1000 dilution: recombinant monoclonal anti‐human Tau (EPR2396[2]; Abcam, cat#: ab109392), rabbit anti‐oligomeric tau antibody T22 (in house, provided by Dr. Rakez Kayed), or mouse anti‐oligomeric tau antibody TOMA2 (in house, provided by Dr. Rakez Kayed). After primary incubation, membranes were washed three times for 10 minutes in 1× TBS/0.05% Tween‐20 and incubated for 1 hour at RT with HRP‐conjugated secondary antibodies (1:5000): anti‐rabbit IgG (Cell Signaling, cat#: 7074, RRID: AB_2099233) for EPR2396(2) and T22; or anti‐mouse IgG (Cell Signaling, cat#: 7076, RRID: AB_330924) for TOMA2. Detection was performed using ECL Western Blotting Detection Reagents (Cytiva, cat#: RPN2209). A standard curve of recombinant tau oligomers (rTauO) was included on each blot to allow comparison across samples.

### 2.4 Morphological analysis of BDTOs by atomic force microscopy

BDTOs were characterized by atomic force microscopy (AFM) as previously described.^19^, ^21^, ^29^, ^30^, ^31^, ^32^ Briefly, samples were prepared by adding 10 µL onto freshly cleaved mica disc (cat# 50‐12; Ted Pella, Inc.) and allowed to adsorb to the surface. Mica was then washed three times with deionized water to remove unbound protein and impurities and then air dried. Samples were imaged with a Multimode 8 AFM machine using a non‐contact tapping method (ScanAsyst‐Air). AFM analyses were performed using the particle analysis tool of the NanoScope Analysis v1.20rl AFM data processing software. All experiments were performed in triplicate.

### 2.5 Proteinase K digestion

The procedure for proteinase K (PK) digestion has been previously outlined.^18^, ^29^, ^33^, ^34^, ^35^ Briefly, molecular‐grade water, Tris HCl, and sodium chloride were mixed in an Eppendorf tube to achieve final concentrations of 100 and 5 mM for the buffers, respectively. BDTOs were then added and incubated with PK at concentrations of 0, 0.5, or 1 µg/mL at 37°C for 1 hour. The reaction was halted by adding 1X lithium dodecyl sufate sample buffer (Invitrogen) and incubating at 95°C for 5 minutes. Afterward, the samples were placed on ice to stop the cleavage process and then loaded onto gel NuPAGE 4% to 12% Bis‐Tris (Invitrogen; cat# NP0336BOX) for sodium dodecyl sulfate polyacrylamide gel electrophoresis or stored at −80°C. Western blotting was performed using Tau5 (1:2000, cat# 806401, BioLegend) and Tau13 (1:1000, cat# 835204, BioLegend) antibodies to detect the digested samples.

### 2.6 Tau RD P301S biosensor cell culture and treatment

Tau RDP301S biosensor cells (ATCC, CRL‐3275) were cultured in Dulbecco's modified Eagle's medium (DMEM) supplemented with 10% fetal bovine serum (FBS), 100 µg/mL penicillin, and 100 µg/mL streptomycin as previously described.^18^, ^33^ Cultures were maintained in a humidified atmosphere of 5% CO_2_ at 37°C. For seeding‐propensity activity assay, the cells were plated on poly‐L‐lysine‐coated coverslips at a density of 1 × 10^5^ cells per well in a 24‐well plate. After 24 hours, cells were transfected with a mixture of BDTOs and Lipofectamine 2000 (cat# 11668‐027; Invitrogen) in Opti‐MEM (cat# 31985‐070 Gibco). Specifically, 0.25 µM BDTOs were combined with 5 µL of Lipofectamine 2000 and diluted in Opti‐MEM to a final volume of 50 µL per well. To assess the seeding specificity of BDTOs, the complexes were pre‐incubated with either an IgG isotype control or the in‐house T22 antibody—which selectively targets tau oligomers and neutralizes BDTO‐related effects—prior to application to cells. The BDTOs/Lipofectamine mixture was incubated at RT for 30 minutes before being added to the cells, bringing the final volume to 300 µL per well. Cells were incubated with the transfection complexes for 24 hours, washed two times with 1X PBS, and fixed with 4% paraformaldehyde for 15 minutes at RT. The slides were then mounted using DAPI Fluoromount‐G mounting media (cat# 0100‐20; SouthernBiotech), dried in the fume hood, and images were acquired using the Olympus confocal microscope (FV1200, Olympus Life Science). A 63X objective was used to capture images from five random fields in triplicate. Volume, area, and mean fluorescent intensity (MFI) analyses were performed using IMARIS software 10.2.0.

### 2.7 Electrophysiology

Six‐month‐old wild‐type mice were anesthetized and euthanized as previously described.^6^ The brain was immediately extracted and sectioned using a Compresstome VF‐300 in an N‐methyl‐D‐gluconate (NMDG)‐based solution to reduce synaptic activity during slicing. Coronal brain slices (300 µm thick), including the hippocampus, were obtained. Throughout the procedure, continuous bubbling with carbogen (95% O_2_ and 5% CO_2_) was maintained to prevent anaerobic conditions. Prior to conducting electrophysiological recordings, half‐brain slices were incubated for 1 hour in a small chamber containing 450 µL of oxygenated holding solution, either with 100 nM AD‐BDTOs, 100 nM NDAN‐BDTOs, derived from four AD and four NDAN subjects, or without BDTOs as a control. This incubation period has been previously demonstrated to be sufficient for primary neurons to internalize BDTOs.^30^ Electrophysiological recordings were performed of the CA1 region of the hippocampus. Excitatory post‐synaptic potentials (EPSPs) were evoked by stimulating Schaffer collateral axons. To begin, slices were stimulated at increasing intensities to generate an input–output curve and determine the stimulus that produced 30% of the maximal response (Figure S1 in supporting information); this intensity was then used for the subsequent two protocols: paired‐pulse stimulation, in which two stimuli were delivered 50 ms apart to assess short‐term synaptic plasticity, and high‐frequency stimulation (HFS), applied at the end of the 10th minute to evaluate long‐term potentiation (LTP).[Fig alz70550-fig-0001]

### 2.8 Cytotoxicity assays

SH‐SY5Y neuroblastoma cells were maintained using DMEM with 10% fetal bovine serum, 4 mM glutamine, penicillin (200 U/mL), and streptomycin (200 µg/mL) in 5% CO2 at 37°C. The medium was replaced every 2 days, and cells were plated in a 24‐well plate having 0.25 × 10^6^ cells per well and grown for 24 hours. To assess BDTOs’ cytotoxicity, the medium was replaced with DMEM containing 1% FBS, and cells were incubated at 37°C in 5% CO_2_ for 1 hour, after which 50 nM, 100 nM, and 250 nM concentrations of BDTOs were applied to identify the optimal concentration for treatment. Cells were observed under a microscope, and the experiment was terminated 3 hours post‐treatment. L‐lactate dehydrogenase (LDH) release was quantified using the Cytotoxicity Detection Kit (LDH; Cat# 11644793001; Roche), according to the manufacturer's protocol. Analysis revealed that 50 nM was the most suitable concentration for subsequent experiments. To evaluate BDTOs’ specificity, BDTOs were pre‐incubated with either an IgG isotype control or the in‐house T22 antibody—which selectively targets tau oligomers and neutralizes BDTO‐related effects—prior to their application to cells. Each treatment was performed in triplicate, and statistical analysis was conducted using one‐way analysis of variance (ANOVA).

To further assess acute cell death by BDTOs, we performed flow cytometry with propidium iodide (PI) staining. Cells were plated in 6‐well plates at a density of 2 × 10⁶ per well and grown overnight. The next day, they were washed twice with complete medium to remove dead cells and treated with 50 nM BDTOs derived from AD or NDAN samples in 2 mL of fresh medium for 4 hours at 37°C in 5% CO_2_. After treatment, the culture medium was collected into 15 mL tubes to preserve floating cells. Adherent cells were incubated with 400 µL of 0.25% Trypsin‐EDTA for 3 minutes at 37°C, after which the collected medium was added to neutralize trypsin. The suspension was mixed and transferred to fresh tubes for centrifugation at 1000 × g for 3 minutes. The resulting pellet was resuspended in 200 µL of 4% paraformaldehyde and incubated for 20 minutes at room temperature. After fixation, cells were centrifuged at 800 × g for 3 minutes, washed three times with PBS, and resuspended in 200 µL of PBS containing 2 µL of PI (2.5 µg/mL). Samples were incubated for 1 minute and immediately the positivity to PI was measured by a Guava EasyCyte flow cytometer (EMD Millipore) and analyzed using FlowJo 10.8.1 software.

### 2.9 Statistical analysis

All data are presented as mean ± standard deviation (SD) or standard error of the mean (SEM), as indicated in figure legends. Group comparisons involving more than two conditions were analyzed using one‐way ANOVA followed by post hoc tests: a Tukey test for pairwise comparisons or a Dunnett test when comparing all treatments to a control group. Two‐tailed unpaired Student *t* tests were used for direct comparisons between two conditions (e.g., AD vs. NDAN in AFM and morphometric analyses). Statistical significance was defined as *P* < 0.05, *P* < 0.005, and *P* < 0.001. Biological replicates (n) are specified in each figure legend. All analyses were performed using GraphPad Prism version 10.0.2.

## 3 RESULTS

**FIGURE 1 alz70550-fig-0001:**
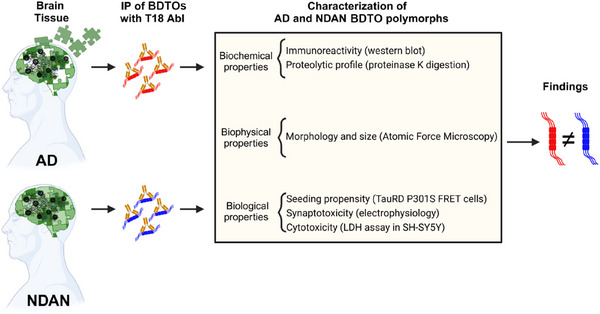
Schematic overview of experimental design. BDTOs were isolated from AD and NDAN brain tissues and characterized using biochemical, biophysical, and biological assays. This integrative approach revealed the presence of two different tau polymorphs in AD and NDAN individuals. AD, Alzheimer's disease; BDTO, brain‐derived tau oligomer; IP, immunoprecipitation; LDH, L‐lactate dehydrogenase; NDAN, non‐demented with Alzheimer's neuropathology.

**FIGURE 2:**
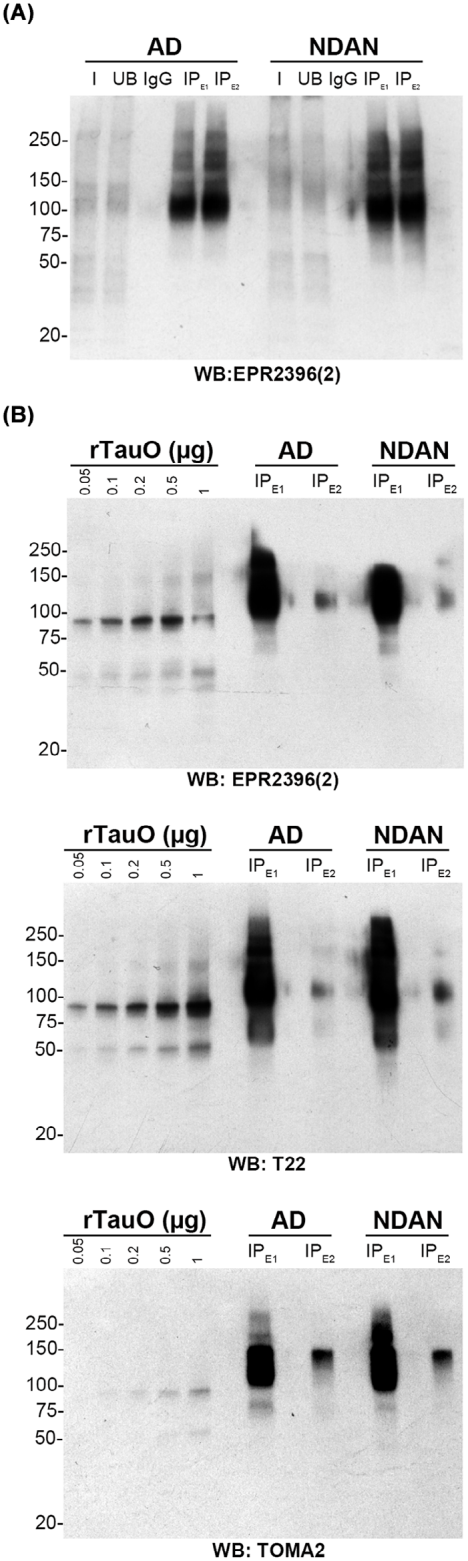
Validation of isolation and immunoreactivity profiles of BDTOs from AD and NDAN individuals. A, Immunoprecipitation of BDTOs from AD and NDAN individuals. Representative western blot showing tau immunoreactivity detected with a recombinant monoclonal anti‐tau antibody in input (I), unbound (UB), immunoglobulin G isotype control (IgG), and immunoprecipitated eluates (IP_E1_ and IPE2) from pooled hippocampal homogenates of age‐matched AD and NDAN individuals. BDTOs were isolated using the in‐house anti‐oligomeric tau antibody T18. Tau enrichment in IP_E1_ and IP_E2_ confirms successful and consistent immunoprecipitation of BDTOs from both groups. B, Immunoreactivity profiles of BDTOs from AD and NDAN individuals. Representative western blot analyses of BDTOs from AD and NDAN individuals (IP_E1_ and IP_E2_), probed with antibodies recognizing total tau (recombinant monoclonal anti‐tau) and oligomeric tau (T22 and TOMA2). Each blot includes a standard curve of recombinant tau oligomers (rTauO) of known concentrations. NDAN‐BDTOs displayed reduced reactivity to the monoclonal anti‐tau antibody, similar reactivity to T22, and increased reactivity to TOMA2 compared to AD‐BDTOs. These patterns were consistent across both IP_E1_ and IP_E2_ fractions, with IP_E2_ showing overall lower levels of immunoreactivity. AD, Alzheimer's disease; BDTO, brain‐derived tau oligomer; IP, immunoprecipitation; NDAN, non‐demented with Alzheimer's neuropathology.

We previously demonstrated that, despite having AD‐like pathology, NDAN brains exhibited key differences from AD patient brains, including preserved synaptic function, distinct proteomic and miRNA profiles, enhanced neurogenesis, improved antioxidant defenses, preserved autophagy, and synaptic resistance to toxic AβO and TauO.^8^, ^9^, ^11^, ^12^, ^14^ To test whether these differences are driven by distinct polymorphs of TauO, we isolated and characterized BDTOs from AD patients and NDAN subjects, as schematically outlined in Figure 1. Homogenates from four age‐matched human *post mortem* hippocampi of AD patients and separately NDAN subjects were pooled and BDTOs were isolated using IP with the in‐house anti‐oligomeric tau antibody T18. The successful isolation of BDTOs was confirmed by western blot (WB), demonstrating their enrichment in both AD and NDAN samples (IP_E1_ and IP_E2_) compared to the input (I), unbound (UB), and isotype control IgG (Figure 2A). Notably, both IP_E1_ and IP_E2_ fractions yielded comparable levels of eluted material, supporting the consistency and reliability of the isolation procedure. After this validation, we further analyzed their biochemical, biophysical, and biological characteristics using the protocols recently reported.^29^

### 3.1 Biochemical/biophysical characterization

**FIGURE 3:**
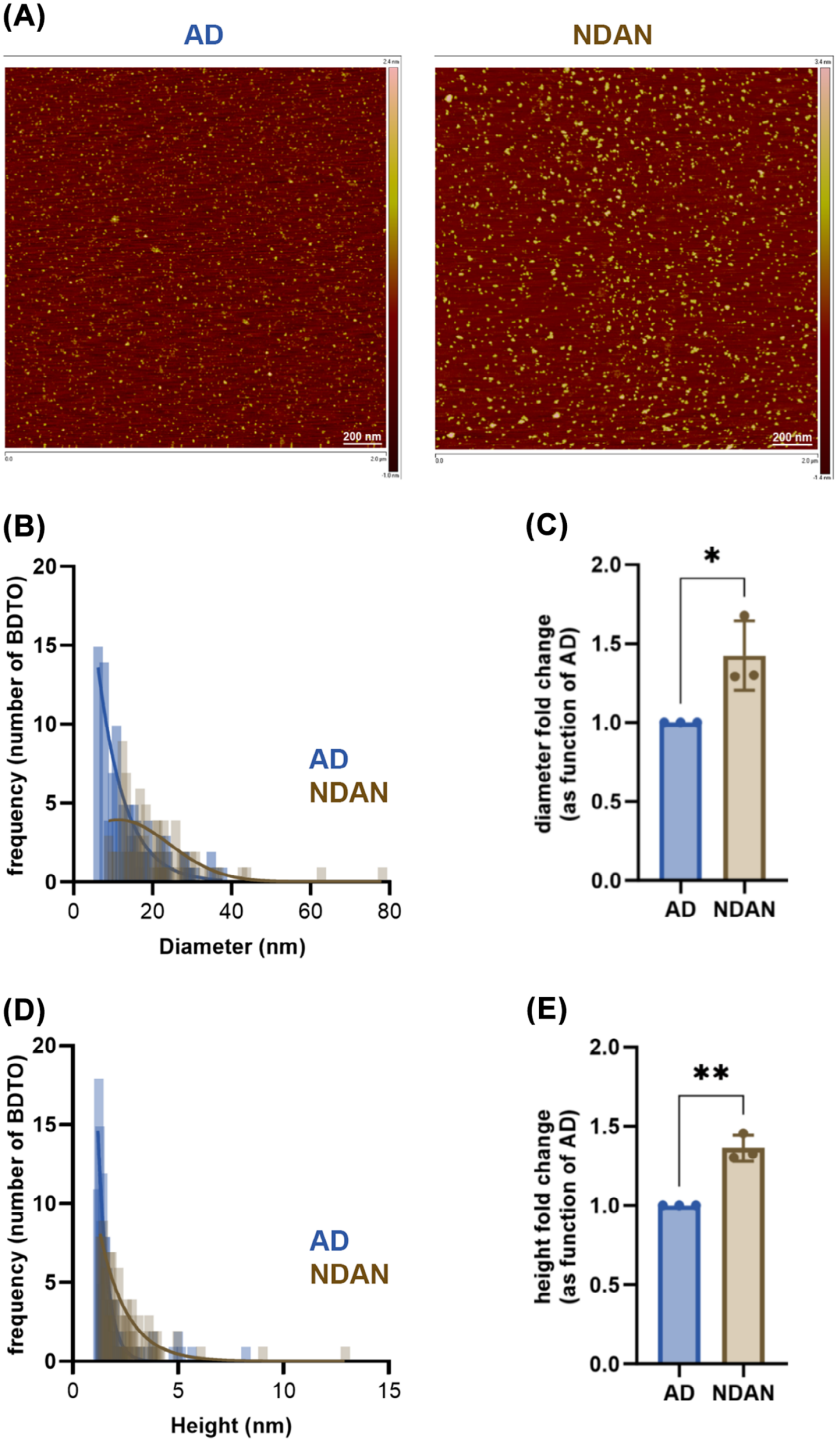
Morphological characterization of BDTOs from AD and NDAN individuals. A, Representative AFM images of BDTOs immunoprecipitated from AD and NDAN hippocampal tissue. B, Distribution histograms and (C) quantification of BDTOs’ diameter shows a significant increase in NDAN compared to AD, represented as fold change relative to AD. D–E, Similar analysis for BDTOs’ height confirms greater vertical dimensions in NDAN‐derived BDTOs. Data indicate that NDAN BDTOs are larger in both diameter and height than their AD counterparts. Data are represented as mean ± standard deviation; biological replicates *n* = 3; P* < 0.05 and **P < 0.005; two‐tailed *t* test. AD, Alzheimer's disease; AFM, atomic force microscopy; BDTO, brain‐derived tau oligomer; IP, immunoprecipitation; NDAN, non‐demented with Alzheimer's neuropathology.

**FIGURE 4:**
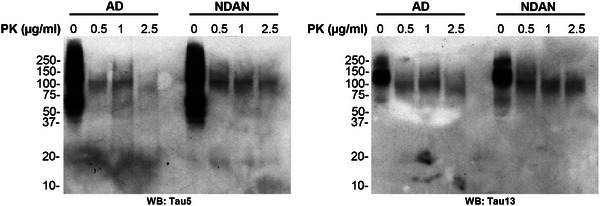
Proteolytic profiling of BDTOs from NDAN and AD individuals. Representative western blot of BDTOs immunoprecipitated from AD and NDAN hippocampal tissue exposed to increasing concentrations of proteinase K (0, 0.5, 1, and 2.5 µg/mL) for 1 hour at 37°C. Blots were probed with Tau5 (left) and Tau13 (right) antibodies to assess proteolytic profiles. NDAN‐BDTO showed greater resistance to degradation compared to AD‐BDTO, suggesting the presence of structurally distinct and more protease‐resistant tau conformers in NDAN. AD, Alzheimer's disease; BDTO, brain‐derived tau oligomer; NDAN, non‐demented with Alzheimer's neuropathology; PK, proteinase K.

First, we analyzed the immunoreactivity of BDTOs from both AD and NDAN subjects to the recombinant monoclonal anti‐tau and to the anti‐oligomeric tau antibodies T22 and TOMA2, respectively, comparing them to a standard curve of recombinant TauO (rTauO) of known concentrations (Figure 2B). We observed distinct immunoreactivity patterns between the two groups, indicating variations in the immunoreactivity profiles across the studied conditions. Specifically, the IP_E1_ fractions exhibited reduced immunoreactivity to the recombinant monoclonal anti‐tau antibody, similar immunoreactivity to the T22 antibody, and increased reactivity to the TOMA2 antibody in NDAN, compared to the reactivity observed in BDTOs from AD. These observations were confirmed in the IP_E2_ fractions, although with overall lower levels of immunoreactivity, supporting the reproducibility of the differential epitope recognition patterns between AD and NDAN BDTOs. Next, we characterized the morphology and size of these BDTOs by subjecting them to AFM (Figure 3). The representative AFM images (Figure 3A) revealed larger BDTOs from NDAN compared to those from AD. We analyzed the distribution of the BDTOs’ population and quantified the differences as fold changes relative to AD BDTOs, for both diameter (Figure 3B, C) and height (Figure 3D, E). Both analyses confirm that NDAN BDTOs exhibit larger diameter and height. These results suggest that the differences in BDTO size may reflect distinct structural characteristics between the two groups. To confirm this, we investigated the proteolytic profiles of BDTOs from both AD and NDAN. In vitro proteolysis is a common approach to study the structure of amyloid proteins by exposing their core regions,^36^, ^37^ revealing distinct protease susceptibility across different aggregates.^38^ It has also been demonstrated that the conformational stability of amplified BDTOs, polymorphic α‐synuclein oligomers, and cross‐seeded recombinant tau oligomers can be distinguished based on their proteolytic profiles.^18^, ^19^, ^29^, ^30^, ^31^, ^33^, ^39^ Here, we exposed AD and NDAN BDTOs to 0.5, 1, and 2.5 µg/mL of PK for 1 hour and evaluated their proteolytic patterns by WB probed with Tau 5 and Tau 13 antibodies (Figure 4). BDTOs from NDAN exhibited greater resistance to proteolysis compared to those from AD, suggesting a lower susceptibility to proteolytic degradation and indicating the presence of different conformations between the two groups.

Together, these data revealed distinct biochemical and biophysical properties of BDTOs from AD and NDAN individuals, including differences in immunoreactivity, size, and protease resistance, suggesting the presence of structurally distinct tau oligomers among the two groups.

### 3.2 Biological characterization

**FIGURE 5:**
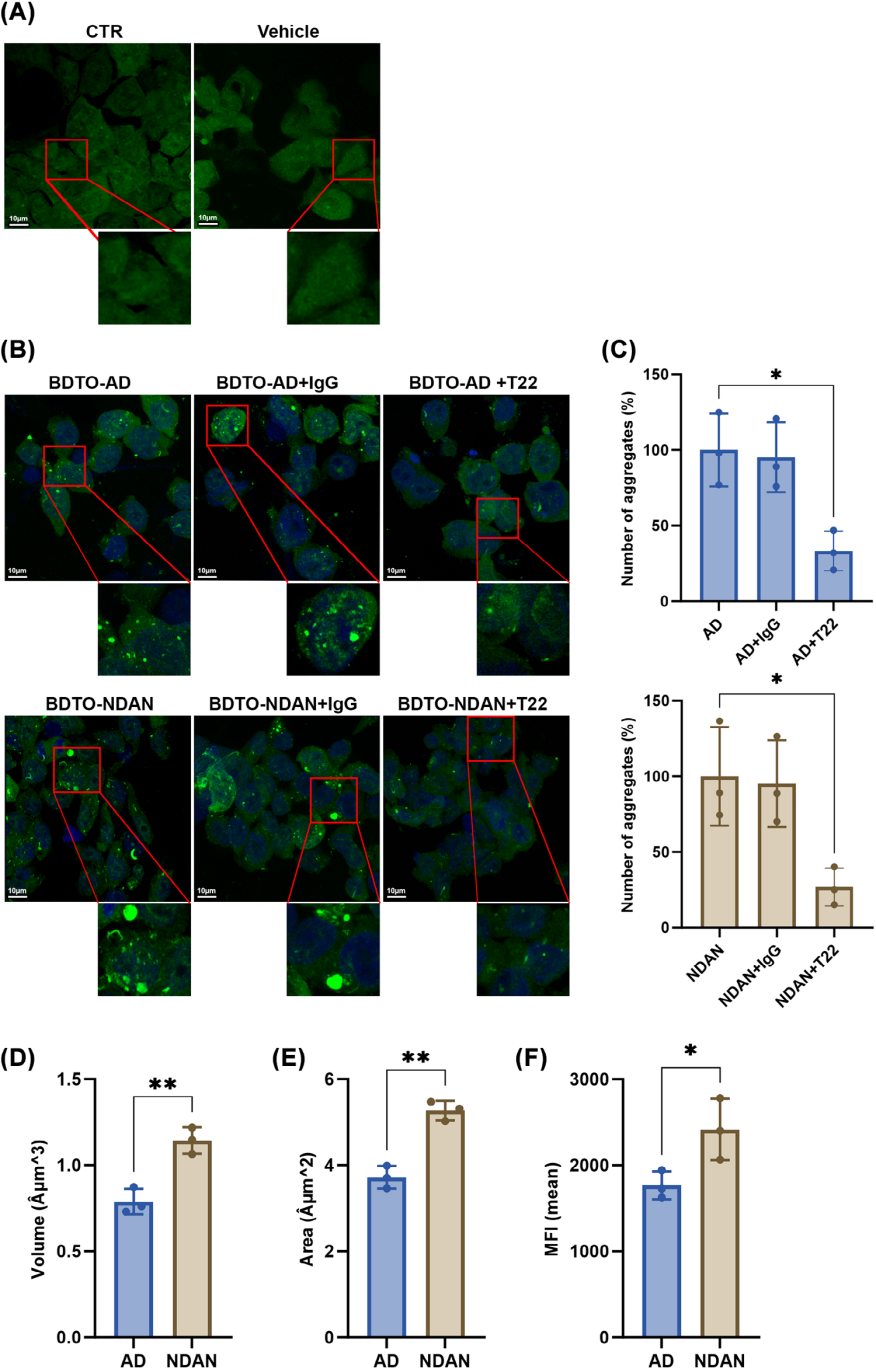
Seeding activity of BDTOs from AD and NDAN. A, Representative FRET images of untreated TauRD(P301S) biosensor cells (CTR) and cells treated with liposome (vehicle), showing no detectable tau aggregation. B, FRET images of biosensor cells treated with 0.25 µM BDTOs from AD and NDAN individuals for 24 hours. Both AD and NDAN BDTOs induced tau aggregation, as evidenced by fluorescent tau aggregates. Cells treated with BDTOs pre‐incubated with an IgG isotype control (middle panel) or BDTOs pre‐incubated with T22 antibody (right panel) are shown. C, Quantification of tau aggregates after BDTOs treatment, comparing the effects of the T22 antibody on seeding activity; data represent the mean ± SD; P* < 0.05; One‐way analysis of variance followed by Dunnett post hoc test. D–F, Morphometric analysis of tau aggregates induced by BDTOs. Volume (D), area (E), and mean fluorescent intensity (MFI; F) were measured for each BDTO‐treated group. Data represent the mean ± SD; biological replicates *n* = 3; P* < 0.05 and **P < 0.005; two‐tailed *t* test. AD, Alzheimer's disease; BDTO, brain‐derived tau oligomer; FRET, fluorescence resonance energy transfer; IgG, immunoglobulin G; NDAN, non‐demented with Alzheimer's neuropathology; SD, standard deviation.

To determine whether BDTOs from AD and NDAN individuals exhibit distinct biological properties, we evaluated their seeding propensity, synaptotoxicity, and cytotoxicity. Seeding activity, which reflects the ability of tau oligomers to induce a conformational change of endogenous tau, thereby initiating aggregation and the formation of toxic oligomers, was assessed using TauRD(P301S) biosensor cells. These cells express a tau repeat domain fused to fluorescent reporters, allowing for the detection of tau aggregation via fluorescence resonance energy transfer (FRET) or tau inclusion formation. FRET specifically distinguishes seeding from accumulation by detecting the recruitment of endogenous tau into nascent aggregates, while tau aggregation itself is confirmed by the formation of tau inclusions. A higher FRET signal indicates greater seeding efficiency.^29^, ^40^, ^41^, ^42^, ^43^ Figure 5 shows that untreated biosensor cells (CTR) and cells treated with the liposome (vehicle) exhibited no detectable FRET signal (Figure 5A), confirming the absence of spontaneous tau aggregation. In contrast, when cells were exposed to 0.25 µM BDTOs from either AD or NDAN individuals for 24 hours, fluorescent tau aggregates became evident, indicating that both types of BDTOs could induce tau seeding (Figure 5B, left panel). To determine whether this effect was specifically mediated by BDTOs, we treated the biosensor cells also with BDTOs pre‐incubated with an IgG isotype control, and BDTOs pre‐incubated with the T22 antibody, which selectively targets and neutralizes tau oligomers. Figure 5B (middle panel) shows that pre‐incubation with the IgG isotype did not alter the FRET signal, suggesting that non‐specific antibody binding had no effect on tau seeding. However, when BDTOs were pre‐incubated with T22 (Figure 5B, right panel), the FRET signal was markedly reduced, indicating that the seeding activity of BDTOs is tau oligomer dependent. Quantification of tau aggregates (Figure 5C) confirmed that immunoneutralization with T22 reduced the seeding by > 50% in both AD and NDAN BDTOs. These findings demonstrated the specificity of BDTO‐driven tau seeding. To evaluate the morphometric parameters of BDTOs in biosensor cells, we quantified the volume (Figure 5D), area (Figure 5E), and mean fluorescent intensity (MFI; Figure 5F) for each BDTO‐treated group. We observed significantly higher values for all three parameters in BDTOs from NDAN compared to those from AD. These data suggest that AD‐ and NDAN‐BDTOs exhibit distinct polymorphs that contribute to their differential seeding capacities, which may underlie variations in synaptic toxicity.

**FIGURE 6:**
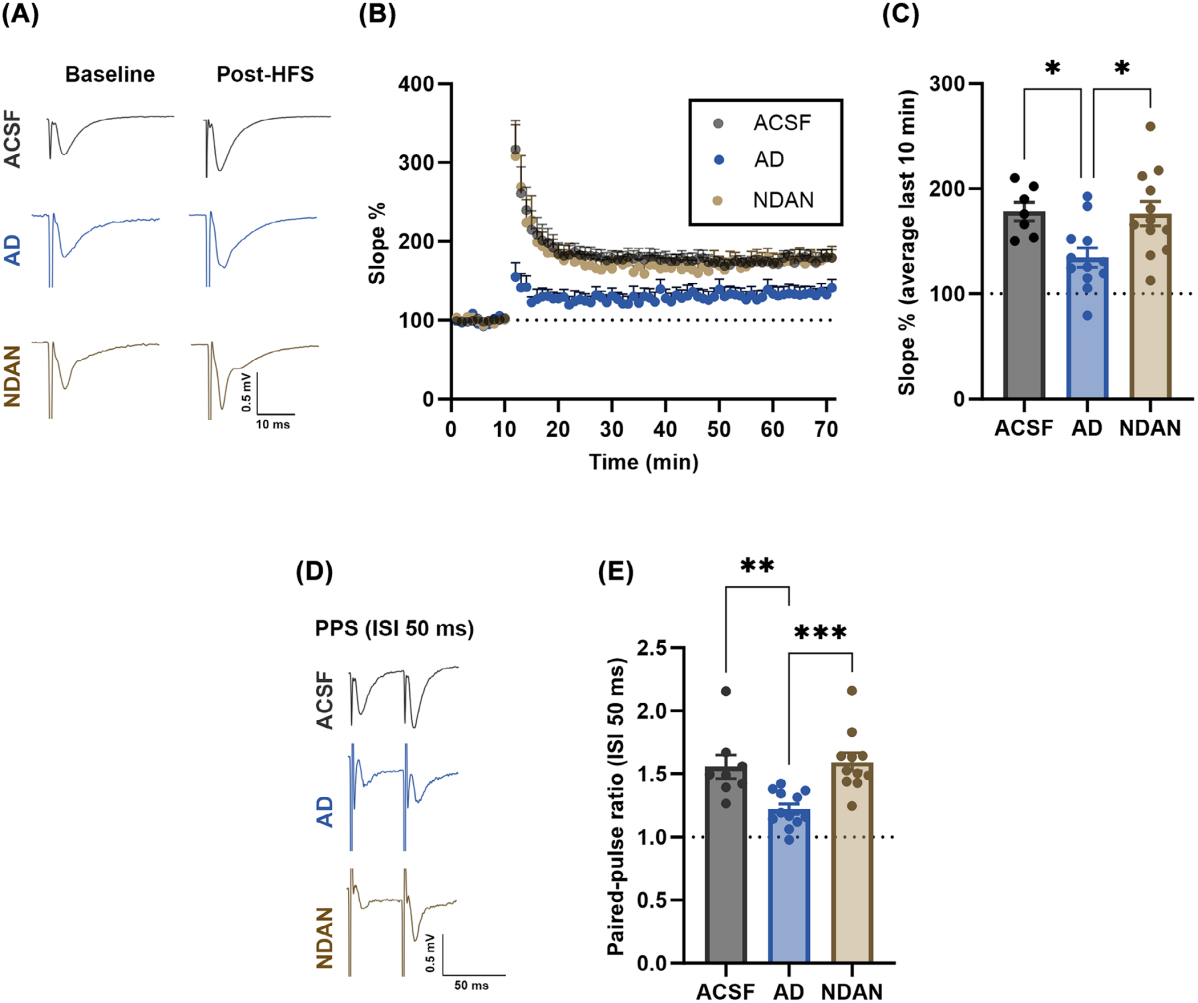
Electrophysiological analysis of synaptic function in hippocampal slices treated with BDTOs from AD and NDAN. A, Representative trace before (baseline) and after (post‐) high frequency stimulation (HFS); (B) Percentage of the EPSP slope. HFS was performed at the end of the 10th minute; (C) Average of the last 10 minutes of the EPSP slope showed that BDTOs from NDAN individuals are less toxic than those from AD; (D) Representative traces of pair‐pulse stimulation with 50 ms interstimulus interval (ISI); (E) paired‐pulse stimulation (PPS) analysis showing impaired paired‐pulse facilitation in slices treated with AD‐BDTOs, indicating dysfunction in short‐term plasticity. Minimum of three different mice each group, each point represents data from a single half‐brain slice (ACSF *n* = 7; AD‐BDTOs *n* = 12; NDAN‐BDTOs *n* = 12, see the Statistical analysis section for criteria of exclusion). Data represent the mean ± standard error of the mean; P* < 0.05, **P < 0.005, ***P < 0.001; one‐way analysis of variance followed by a Tukey post hoc test. ACSF, artificial cerebrospinal fluid; AD, Alzheimer's disease; BDTO, brain‐derived tau oligomer; EPSP, excitatory post‐synaptic potential; HFS, high‐frequency stimulation; IgG, immunoglobulin G; NDAN, non‐demented with Alzheimer's neuropathology; SD, standard deviation.

To investigate the toxicity profiles, hippocampal slices from wild‐type C67BL/6J mice were treated for 1 hour with either artificial cerebrospinal fluid (ACSF) or 100 nM of BDTOs from both AD and NDAN, before conducting electrophysiological field recordings in the hippocampal Shaffer collateral–CA1 synaptic pathway. LTP was then assessed to evaluate the functional impact of these distinct BDTOs on synaptic plasticity (Figure 6). Basal synaptic transmission did not show differences within the experimental groups (Figure 6A), while LTP profiles and analysis showed a repressed LTP only in the slices treated with AD BDTOs (Figure 6B, C). To determine dynamic properties of synaptic transmission, specifically short‐term plasticity, we performed paired‐pulse stimulation (PPS) that revealed a significant impairment of paired‐pulse facilitation by the treatment with BDTOs from AD, compared to NDAN and ACSF (Figure 6D, E). These data suggest that BDTOs from AD are more synaptotoxic than those from NDAN, disrupting both postsynaptic potentiation and presynaptic neurotransmitter release dynamics. Although we did not directly monitor neuronal hyperexcitability markers such as spontaneous firing or epileptiform discharges, the synaptic dysfunction observed here may reflect early alterations in excitation/inhibition (E/I) balance, which has been documented in human AD cortex.^44^, ^45^

**FIGURE 7:**
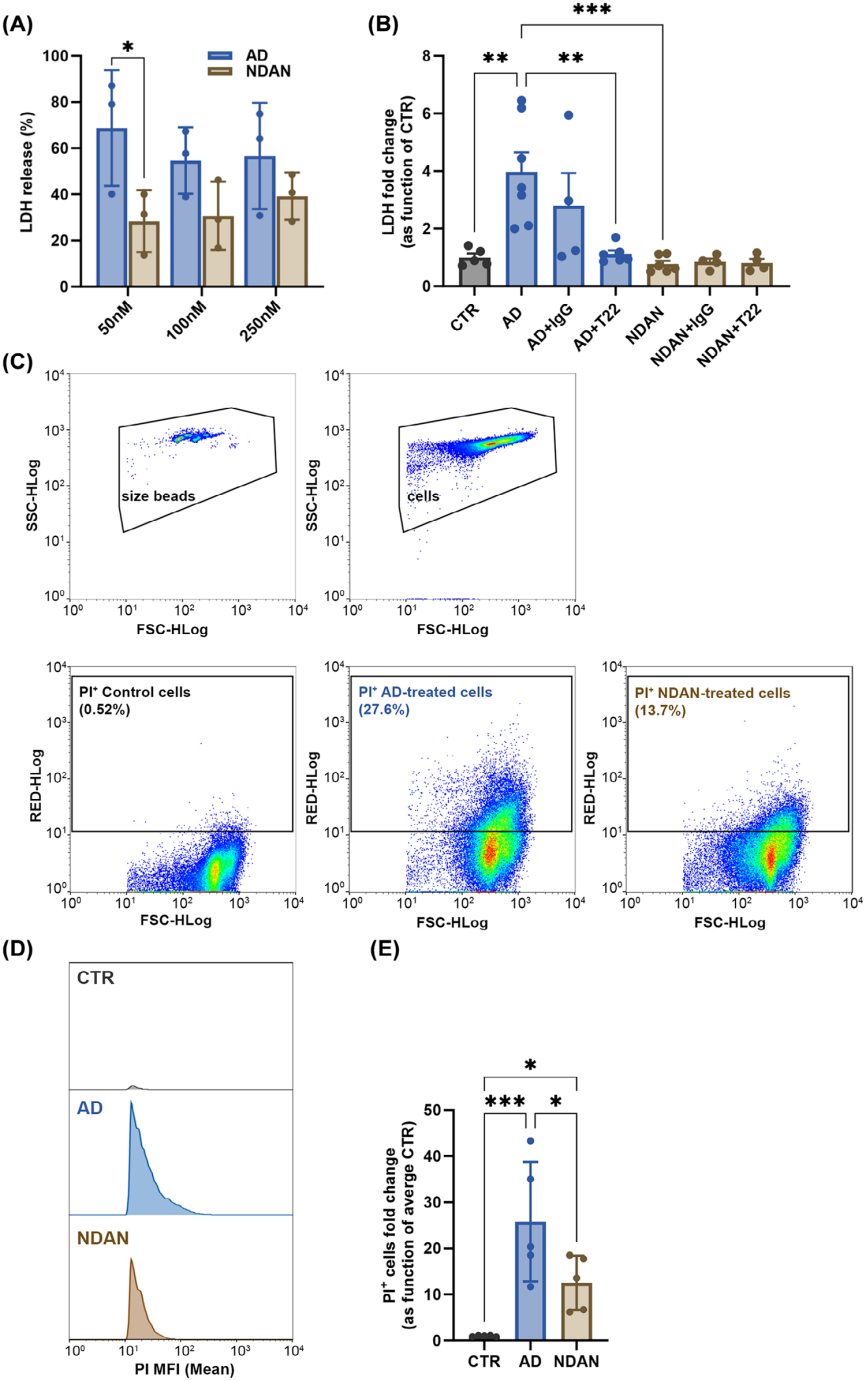
Cytotoxicity analysis of BDTOs from AD and NDAN individuals. A, LDH release from SH‐SY5Y cells treated for 4 hours with increasing concentrations (50, 100, and 200 nM) of BDTOs from AD and NDAN individuals to determine the optimal concentration for the assay. Data represent the mean ± SD; biological replicates, *n* = 3; *P < 0.05; one‐way ANOVA followed by a Tukey post hoc test. B, SH‐SY5Y cells were treated with the selected concentration (50 nM) of BDTOs, BDTOs pre‐incubated with IgG isotype control, or BDTOs pre‐incubated with T22 antibody to assess the specificity of the cytotoxic effect. Data represent the mean ± SD; each dot represents a biological replicate (CTR, *n* = 5; AD, *n* = 7; AD+IgG, *n* = 4; AD+T22, *n* = 6; NDAN, *n* = 6; NDAN+IgG, *n* = 4; NDAN+T22, *n* = 4); **P < 0.005, ***P < 0.001; one‐way ANOVA followed by a Tukey post hoc test. C, Representative plots showing the gating strategy for cytofluorimetric analysis of propidium iodide (PI) incorporation. The upper left panel shows bead‐based gating; the upper right panel shows gating for SH‐SY5Y cells. Lower panels display the percentage of PI‐positive (PI⁺) cells for control, AD‐treated, and NDAN‐treated groups based on these gates. D, Histograms of mean fluorescence intensity (MFI) of PI⁺ SH‐SY5Y cells for all treatment conditions, generated from combined data across all biological replicates (n) to illustrate overall distribution patterns. E, Quantification of MFI values shown in (D), expressed as fold change relative to untreated control cells. Data represent the mean ± SD; biological replicates, *n* = 5; *P < 0.05, **P < 0.005, ***P < 0.001; one‐way ANOVA followed by a Tukey post hoc test. AD, Alzheimer's disease; ANOVA, analysis of variance; BDTO, brain‐derived tau oligomer; IgG, immunoglobulin G; LDH, L‐lactate dehydrogenase; NDAN, non‐demented with Alzheimer's neuropathology; SD, standard deviation.

After assessing the effects of BDTOs on the synapses, we next investigated their cytotoxicity. To do so, we treated human neuroblastoma SH‐SY5Y cells with BDTOs for 4 hours and measured LDH release, which is a widely used marker of membrane damage and cell lysis.^46^ SH‐SY5Y cells were chosen based on previous studies demonstrating their suitability as model for studying tau‐induced toxicity and for assessing LDH release as an indicator of cytotoxicity. This approach is supported by evidence that tau oligomers can compromise cellular membrane integrity, leading to increased permeability and subsequent leakage of intracellular components such as LDH.^47^, ^48^ Initially, we tested the LDH release in cells treated with three different concentrations of BDTOs (50, 100, and 200 nM) derived from both AD and NDAN individuals. This was done to determine which concentration elicited the most significant cytotoxic effect (Figure 7A). Based on these preliminary measurements, we identified 50 nM as the optimal concentration of BDTOs, as it induced measurable cytotoxicity without overwhelming the assay. Specifically, we observed an increase in LDH release of ≈ 4‐fold relative to untreated cells (CTR) in cells treated with AD‐BDTOs compared to those treated with BDTOs from NDAN individuals. To further confirm that the observed cytotoxicity was specifically mediated by BDTOs, we conducted additional treatments using BDTOs pre‐incubated with an IgG isotype control and BDTOs pre‐incubated with the T22 antibody, following similar experimental conditions used in the FRET assay. The data presented in Figure 7B reveal varying levels of LDH release across different treatment conditions. The highest LDH release (≈ 4‐fold relative to CTR) was observed in cells treated with BDTOs from AD and in cells treated with BDTOs from AD pre‐incubated with IgG isotype control, both indicating significant cytotoxicity compared to CTR cells. This effect was abolished when the BDTOs from AD were neutralized by pre‐incubation with the T22 antibody. In contrast, cells treated with BDTOs from NDAN individuals, whether neutralized or not, showed similar levels of LDH release that were comparable to the CTR, suggesting no significant cytotoxicity in these conditions. Furthermore, cytofluorimetric analysis of propidium iodide‐positive (PI⁺) SY5Y‐SH cells was performed to assess cell membrane integrity after treatment with BDTOs from either AD or NDAN individuals. PI is a membrane‐impermeable dye that selectively stains cells with compromised membranes, serving as a marker of late‐stage cytotoxicity. Comparison to untreated cells revealed varying levels of PI incorporation across treatment conditions, with the highest PI positivity observed in cells treated with AD‐BDTOs, indicating their greater cytotoxic potential than NDAN BDTOs (Figure 7C‐E).

## 4 DISCUSSION

This study demonstrated that tau oligomers isolated from the brains of AD and NDAN individuals exhibit distinct structural, biochemical, and functional properties, with important implications for their cytotoxic and synaptotoxic potential. Our findings suggest that tau polymorphisms play a significant role in disease resilience, with tau oligomers from NDAN individuals exhibiting structural differences associated with lower neuronal toxicity, contributing to cognitive resilience despite the presence of AD‐like pathology. These differences are reflected in key biochemical, biophysical, and functional traits, including immunoreactivity, protease resistance, size, aggregation dynamics, and toxicity, offering novel insights into the differential toxicity of tau oligomers in AD pathogenesis.

The biochemical and biophysical characterization of BDTOs revealed key structural differences between AD and NDAN individuals. Immunoreactivity assays indicated that NDAN‐BDTOs display distinct conformational epitopes, suggesting potential differences in folding or aggregation states. Furthermore, AFM analyses showed that NDAN‐BDTOs exhibit larger oligomeric assemblies compared to AD‐BDTOs. Because tau oligomer size has been linked to toxicity, these differences could underlie the reduced pathogenicity observed in NDAN individuals.^49^, ^50^ The observed increase in diameter and height, together with the greater resistance to PK digestion, strongly suggests that NDAN BDTOs adopt a more compact and protease‐resistant structure. Such compactness could shield protease‐sensitive regions, reflecting a more ordered conformation compared to AD‐BDTOs. The larger size of NDAN‐derived oligomers may be indicative of a more stable tau conformer, which could influence its ability to aggregate and interact with synaptic components, providing a potential explanation for the cognitive resilience observed in NDAN individuals. Previous studies have demonstrated that smaller, soluble tau oligomers exhibit greater neurotoxic potential than larger aggregates, as they more readily impair synaptic function, induce mitochondrial dysfunction, and promote neurodegeneration.^6^, ^51^, ^52^ In addition, PK digestion assays indicated that NDAN‐BDTOs are more resistant to proteolysis, suggesting a more stable conformation that may influence their biological activity. This structural stability may also reduce the availability of toxic oligomeric surfaces or expose different biochemical properties that lower their synaptotoxicity. This protease resistance may be a key factor in the reduced cytotoxicity of NDAN‐BDTOs, as it could result in more stable, less‐toxic tau conformers that are less likely to disrupt cellular function. Together, these findings support the hypothesis that there is structural heterogeneity of NDAN‐BDTO compared to AD‐BDTO, suggesting the existence of a distinct TauO polymorphs underscoring cognitive resilience.

Beyond structural differences, we observed functional disparities in the seeding capacity and neurotoxic potential of AD‐ and NDAN‐BDTOs. Seeding activity—reflecting the prion‐like ability of tau oligomers to recruit endogenous tau into misfolded aggregates—was assessed using FRET‐based biosensor assays. It is important to distinguish that seeding refers to the initiation of misfolding, while aggregation describes the subsequent growth and accumulation of tau assemblies. Previous studies have shown that both the efficiency of seeding and the physical properties of aggregates critically influence toxicity. While enhanced seeding promotes the pathological spread of tau, larger and more stable aggregates may be less neurotoxic, likely due to decreased solubility and reduced interaction with cellular membranes.^53^ Additionally, faster aggregation kinetics have been associated with heightened cellular stress and degeneration,^54^ underscoring the impact of tau dynamics on disease progression. Structural differences among tau oligomers, as seen between AD‐ and NDAN‐BDTOs, are known to influence these behaviors, contributing to the heterogeneity observed across tauopathies.^55^ In this study, both AD‐ and NDAN‐BDTOs demonstrated the ability to seed tau aggregation in biosensor cells, but NDAN‐derived aggregates appeared larger than those induced by AD‐BDTOs. Given identical treatment conditions, this suggests that although both types can initiate tau misfolding, the subsequent aggregation dynamics differ, with NDAN‐BDTOs promoting the formation of larger, potentially more stable assemblies. These differences in seeding and aggregation behavior may underlie the reduced pathogenicity observed in NDAN individuals and support the notion that not all tau seeds exert the same biological impact.

In line with these observations, electrophysiological recordings demonstrated that AD‐BDTOs impaired hippocampal LTP, whereas NDAN‐BDTOs had no significant effect. AD‐BDTOs also disrupted paired‐pulse facilitation, suggesting deficits in presynaptic neurotransmitter release, consistent with previous studies implicating tau oligomers in synaptic dysfunction.^3^, ^6^, ^49^ The absence of synaptotoxic effects in NDAN‐BDTOs suggests that their structural properties may confer resilience to tau‐mediated synaptic impairments. Although our electrophysiological approach focused on synaptic plasticity rather than network excitability, our findings align with evidence from human studies showing synaptic‐level E/I imbalances and circuit hyperactivity in AD.^44^, ^45^ Future work will aim to directly assess whether distinct tau polymorphs contribute differentially to neuronal excitability.

In agreement with electrophysiological data, cell viability assays revealed that AD‐BDTOs induce significantly greater cytotoxicity compared to NDAN‐BDTOs. Increased LDH release and PI staining indicate that AD‐BDTOs promote membrane permeability and neuronal damage, hallmarks of tau oligomer toxicity.^47^, ^48^, ^56^ Given that LDH release is an acute marker of membrane damage and cell lysis, we assessed its levels in human neuroblastoma SY5Y‐SH cells after a 4 hour treatment with BDTOs. This timeframe allowed us to capture early disruptions in membrane integrity, providing insight into the direct cytotoxic effects of tau oligomers. Consistently, AD‐BDTOs triggered significantly greater LDH release compared to NDAN‐BDTOs, reinforcing that differences in tau oligomer structure may modulate their ability to disrupt cellular membranes and contribute to neuronal damage. Additionally, BDTOs derived from patients with AD and other tauopathies have been shown to promote internalization, exacerbating membrane permeability and contributing to cellular dysfunction and neuronal damage.^30^ The lower cytotoxicity of NDAN‐BDTOs suggests that they either possess reduced intrinsic toxicity, are less capable of interacting with and disrupting cellular membranes, or are more susceptible to clearance mechanisms, potentially contributing to the cognitive resilience observed in NDAN individuals.^8^, ^9^, ^11^, ^14^ In addition, in our previous work, we demonstrated that the ability of NDAN individuals to maintain cognitive integrity despite substantial AD pathology is linked to preserved synaptic integrity and maintenance of key synaptic proteins such as PSD‐95 and synaptophysin.^12^ Furthermore, we also reported remodeling and preservation of dendritic spines in NDAN brains.^14^ These observations support the idea that tau oligomers in NDAN cases may engage distinct biological pathways compared to those in AD.

Taken together, these findings provide compelling evidence that tau oligomer heterogeneity plays a critical role in determining disease progression and resilience. Indeed, the presence of a structurally and functionally distinct tau polymorph in NDAN as reported here, is, to the best of our knowledge, the first observation of a non‐toxic TauO polymorph underscoring cognitive resilience in AD. Understanding the mechanisms underlying this differential toxicity may open new therapeutic avenues aimed at stabilizing less toxic tau conformers or enhancing the clearance of pathogenic species. Future studies should further explore the molecular determinants of these differences, including post‐translational modifications, interaction with chaperones, and the role of proteostasis networks in shaping TauO profiles, as well as their differential capacity to induce neuroinflammatory responses in vivo. Ongoing studies in our lab involving intracerebroventricular injection of AD and NDAN tau oligomers aim to assess glial activation profiles and their contribution to synaptic vulnerability or resilience. Our study of tau polymorphisms underscores the need for a more comprehensive approach to AD research, one that considers not only the presence of tau aggregates but also their structural and functional diversity. These insights have the potential to refine our understanding of AD pathogenesis and resilience, paving the way for targeted interventions that preserve cognitive function despite tau pathology.

## AUTHOR CONTRIBUTIONS

All authors made substantial contributions to the development of the study. Michela Marcatti performed the analyses of BDTO morphology, size, seeding propensity, and cytotoxicity, and drafted the manuscript. Batbayar Tumurbaatar carried out BDTO isolation and biochemical characterization, including immunoprecipitation, western blotting, and proteinase K digestion; and contributed to drafting the methods section of the manuscript. Wen‐Ru Zhang contributed to the western blot experiments and cytotoxicity. Pietro Scaduto and Jutatip Guptarak conducted synaptotoxicity analyses using electrophysiology. Shrinath Kadamangudi and Regan J. Schuetze assisted with BDTO isolation and western blotting. Anna Fracassi contributed to the study design and statistical analysis. Rakez Kayed provided the protocols for BDTO isolation and characterization established in his laboratory. Giulio Taglialatela conceived, designed, and funded the study and provided final approval of the manuscript. All authors reviewed and approved the final version of the manuscript.

## CONFLICT OF INTEREST STATEMENT

The authors declare no conflicts of interest. Author disclosures are available in the supporting information.

## CONSENT STATEMENT

Informed consent was obtained from all participants prior to their enrollment in the studies at the AD Center (ADC) at OHSU, in Portland, OR (USA).

## Supporting information



Supporting Information

Supporting Information
