# Supplementary figures and images for: A brain‐derived tau oligomer polymorph is associated with cognitive resilience to Alzheimer's disease
Source: Alzheimers Dement. 2025 Aug 18;21(8):e70550. doi: 10.1002/alz.70550 (PMC12359067; doi:10.1002/alz.70550)

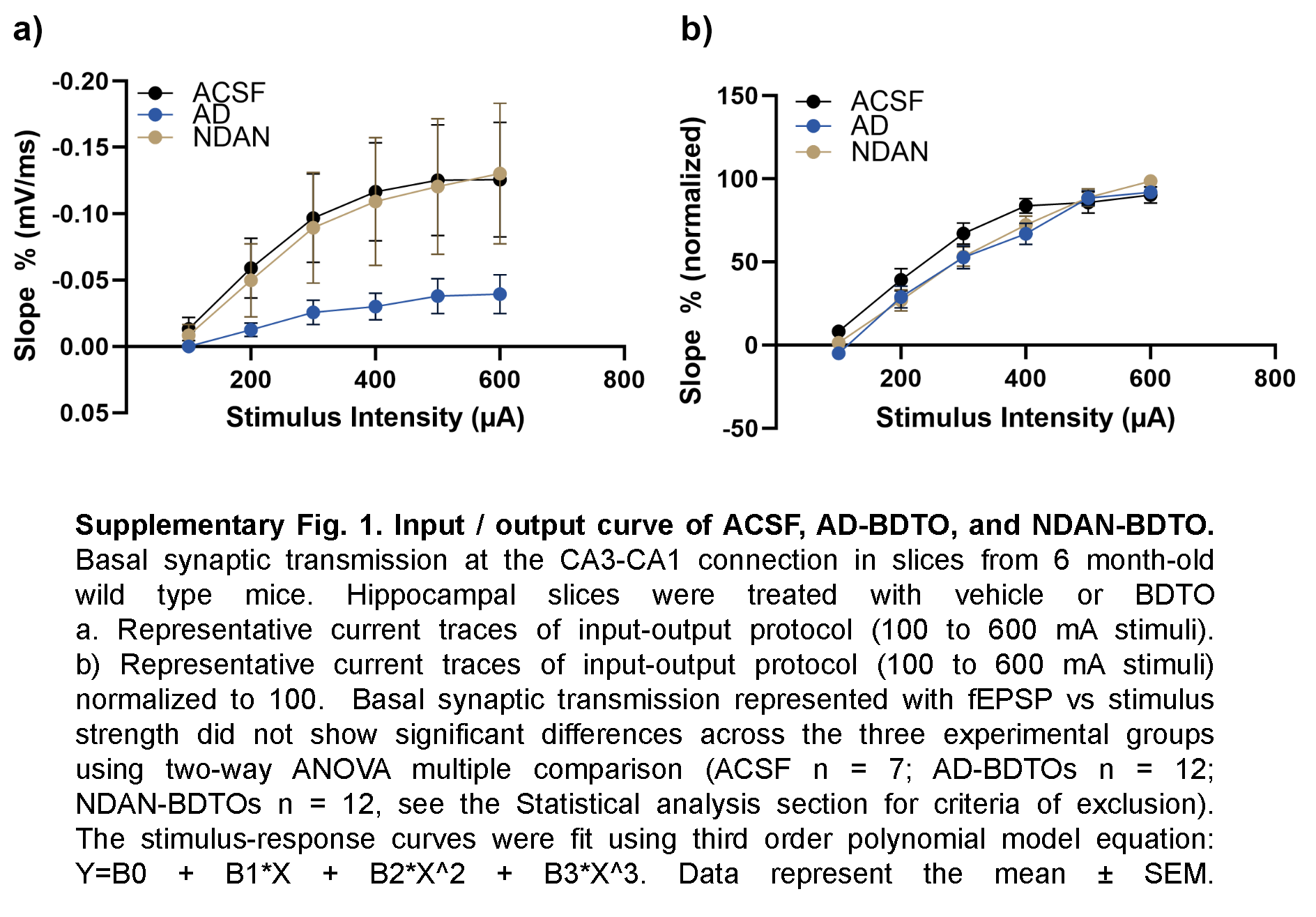

Supplement: Supplementary file 1 — Supporting Information [file ALZ-21-e70550-s002.tif]
